# Acute Glanders

**Published:** 1897-09

**Authors:** R. L. Denman

**Affiliations:** Durst, Texas


### Correspondence.

---

### Acute Glanders.

---

DURST, TEXAS, August 9, 1897.

*Editors Texas Medical Journal:*

Having treated a case of acute glanders, a disease which is so rarely met with, in the human subject, and its symptoms and course being so different from what we might expect from reading text-books, I think it is my duty to report it, hoping it will be of advantage and interest to all who read it.

Supposing all are familiar with the definition, etiology, symptomatology, prognosis, etc., of acute glanders, and wishing to occupy not too much space, I will only give the history, symptoms, course and termination of this case.

On March 7, 1896, I was called to see Mr. A., aged about 39, temperate habits, no hereditary taint of any kind. Had previously had fair average health. At times he said he was subject to muscular and arthritic pains; had an attack of continued fever the year before. I found him with a well marked chill and all the symptoms peculiar to a malarial attack; temperature 101° F., pulse 80. Gave him a cardiac stimulant and an antipyretic. In a few minutes all the chilly sensation disappeared; diaphoresis soon set in, and in two hours he had no fever, and said he was feeling as well as ever. There being more malaria here than anything else, and this case acting just as an intermittent malarial attack does, I at once proceeded to treat him for malaria, notwithstanding I knew he had been treating some of his stock that had died of what proved to be glanders. But as I could not find any lesion that looked suspicious of his being inoculated with the virus of glanders, there being no catarrhal symptoms, and the disease being so uncommon in man, I did not once suspect it was glanders, until about the end of the second week.

March the 8th, the patient had another well marked chill, followed by apyrexia and diaphoresis, near the same time of the day as on the preceding, with same general symptoms of malaria, to-wit: muscular soreness, severe frontal headache, and he complained of acute pains in the back, all of which disappeared with the subsidence of the fever. Believing I had not the general secretory and excretory system well aroused, a mercurial purgative was given and followed with heavier doses of quinine than before, with the full assurance of warding off the next paroxysm; but on the 9th, about one hour later, he had another paroxysm as above, not ameliorated in the least, nor exacerbated. I now gave eight-grain doses of quinine in solution, every three hours, till four doses were given, then directed it to be given every four hours. The paroxysm was missed, as I thought, as I stayed with him two hours past the hour, and he seemed to be all right; but four hours later I was called to see him again. On arriving, I found that reaction from another chill had just set in. The bowels were very much constipated, notwithstanding the repeated mercurial, hence I repeated the mercurial purge with better effects. He was somewhat nauseated, and vomited for the first time during this paroxysm. I kept up the quinine, and returned next morning and found patient in good spirits; he did not complain of being prostrated; appetite good. His tongue was heavily coated with a thick white fur, and appeared to be larger than usual, and thickened as is usual in malarial cases. At this time he called my attention to a small innocent looking furuncle on the middle anterior aspect of the right forearm. It was about the size of a small bird egg, somewhat pointed, with but slight inflammatory base; no œdema or perceptible enlargement of lymphatic glands. He did not complain of its paining, more than would a small boil. I told him I would open it in a few days. The sclera in the outer canthus of the right eye was somewhat injected, and showed an extravazation of blood, such as occurs from a severe blow near the eye, but it was not painful nor sore. I kept up the anti-malarial treatment, hoping sooner or later to stop the daily recurring paroxysms, but without effect. On the 10th, the fever assumed a remittent form; would rise to 101° in the evening, sometimes 103°, but by 12 o'clock at night it would fall to 99½ or 100°, and remain so till the next evening, and then rise again. This continued several days, then it was accompanied by rheumatic pains.

It would require too much space to give all the details of the case; suffice to say that at this stage he had a slight rigor, after which the symytoms of rheumatism were constant. Temperature reached 104°, acid perspiration, acid urine, soreness, pain, and swelling in the sterno-clavicular region. This remained only a few days and shifted to the wrist, then to the ankle, and so on. The furuncle finally broke down and was covered with a hard dark scab. It showed no tendency to get either better or worse. There were still no local symptoms of the disease only those above stated, but I had by this time fully made up my mind it was a case of glanders, but did not of course express my suspicions. Patient was still cheerful, and had not failed in strength; his appetite was still reasonably good; pulse not going over 100 up to the third week. But the pain and restlessness from the increase of fever after this time, from 101° to 103° or 104° soon began to tell upon his strength, and he gradually grew weaker, and became less hopeful, and just four days before the appearance of the characteristic local symptoms, such as the pustular eruption of the skin and the catarrhal symptoms of the disease (but the lymphatic system did not show any evidence of the disease as described in Quain's Dictionary of Medicine), he slowly drifted into a semi-delirious state; this being the middle of the fourth week of his illness. Upon the appearance of local characteristic symptoms, I thought every one ought to be warned of danger, and accordingly steps were taken to prevent others from being inoculated.

I summoned my brother, Dr. A. M. Denman, and Dr. Largent, of Lufkin, Texas, and both agreed with me in the diagnosis, glanders. Then Dr. C. C. Brown, of Tyler, Texas, veterinary surgeon, was summoned and confirmed the diagnosis, having personally seen a few cases in man. He secured a specimen from a pustule and reported later, finding the bacilli characteristic of this disease.

Patient died April 14th, of exhaustion, and in a state of profound coma.

Now I want to ask: Could I have made a diagnosis of the disease earlier with nothing but the constitutional symptoms and history to guide me, in the absence of the characteristic lesion of inoculation and local symptoms? There is no doubt the innocent looking furuncle on the arm was the seat of the poison whence it entered the system. It did not show what the authorities claim. The "erysipelatous redness," for instance. It did not get larger, nor grow worse, nor assume a chancroial appearance; nor did it discharge dirty sanious and offensive matter, but rather the opposite of these conditions; hence I was misled perhaps.

Nothing remained to show for it after the 10th or 12th day, only an ulcer, which looked somewhat like a sore from vaccination just getting ready to shed the scab, until the eruption appeared; then it took on the same identical picture and run the same course as vaccination. At first it became red and inflamed, as did the spots on the body which are said to be characteristic of the disease; first like flea-bites, then elevated (papular), then vesicular, and ultimately an inflamed pustule. During the course of the disease every symptom was well marked, except there was no involvement of the lymphatic system.

In conclusion, I will say that at the time the fever assumed the remittent form, I became satisfied of having produced profound cinchonism, but without any amelioration of the symptoms; therefore, I discontinued the anti-malarial treatment, and adopted a purely symptomatic one, and continued it through the rheumatic course.

It is needless to say that had I known at first that the disease was glanders, the result would have been the same, as all know there is no remedy for its cure. Its treatment, as given in the books, being just about the same as I used (symptomatic). The treatment undoubtedly prolonged the life of the patient two to three weeks.

I regret, however, that the disease did not show its characteristics sooner, as in that event I could have made better observation of its course and symptoms.

In future, if I should meet with a case with the same history, and running the same or any other course, and could find anything on the body that looked like a sore, either inflammatory or non-inflammatory, I would diagnose glanders, and treat the sore by local application. It is needless to say that this case ran an acute course of farcy, with the exception that it did not show itself in the lymphatic system, and just before death, assumed all the local manifestations of acute glanders, as it sometimes does in the horse. I know now the constitutional symptoms were from the effects of the glander vivus, nevertheless, as you see, it did not altogether tally with the description in the text-books.

Fraternally,

R. L. Denman, M. D.

